# Atrial Cardiomyopathy: Pathophysiology and Clinical Consequences

**DOI:** 10.3390/cells10102605

**Published:** 2021-09-30

**Authors:** Andreas Goette, Uwe Lendeckel

**Affiliations:** 1 Department of Cardiology and Intensive Care Medicine, St. Vincenz Hospital, 33098 Paderborn, Germany; 2 MAESTRIA Consortium/AFNET, 48149 Münster, Germany; 3 Institute of Medical Biochemistry and Molecular Biology, University Medicine Greifswald, 17475 Greifswald, Germany; uwe.lendeckel@med.uni-greifswald.de

**Keywords:** atrial fibrillation, molecular biology, oxidative stress, cardiomyopathy, inflammation

## Abstract

Around the world there are 33.5 million patients suffering from atrial fibrillation (AF) with an annual increase of 5 million cases. Most AF patients have an established form of an atrial cardiomyopathy. The concept of atrial cardiomyopathy was introduced in 2016. Thus, therapy of underlying diseases and atrial tissue changes appear as a cornerstone of AF therapy. Furthermore, therapy or prevention of atrial endocardial changes has the potential to reduce atrial thrombogenesis and thereby cerebral stroke. The present manuscript will summarize the underlying pathophysiology and remodeling processes observed in the development of an atrial cardiomyopathy, thrombogenesis, and atrial fibrillation. In particular, the impact of oxidative stress, inflammation, diabetes, and obesity will be addressed.

## 1. Introduction

In 1995, Allessie’s group introduced the term “atrial electrical remodeling”. His fundamental study showed that atrial fibrillation (AF) causes shortening of the atrial action potential and that, thereby, AF begets AF [1]. In the following year, Goette et al. reported about the underlying electrophysiological mechanism of atrial electrical remodeling [2]. In their model, autonomic blockade was induced with infusion of atropine and propranolol. Thereafter, high-rate atrial pacing was applied for a total of 7 h. Atrial effective and absolute refractory periods (ERP and ARP) were determined at least once an hour during 7 h of pacing. Of note, the infusion of verapamil abolished electrical remodeling. To determine whether the verapamil effect could be overcome by hypercalcemia, experiments were repeated with a combination of verapamil plus administration of calcium gluconate at the end of the study. Interestingly, reversal of the verapamil effect was seen suggesting that the anti-remodeling effect of verapamil is mediated by L-type calcium channel blockade, rather than a nonspecific drug effect. Furthermore, the effect of glibenclamide infusion was tested [2]. Glibenclamide per se did affect the magnitude or time course of electrical remodeling. Microscopic analyses of atrial tissue were normal in all experimental groups. There were no contraction bands, interstitial edema, cellular infiltration, or nuclear pyknosis [2]. However, electron microscopic studies showed mitochondrial swelling after 7 h of rapid atrial pacing. The mitochondrial swelling was associated with lysis of the mitochondrial cristae. Abnormalities of the transverse tubular system or of the sarcoplasmic reticulum were not seen. Verapamil treatment could prevent structural mitochondrial changes [2]. Thus, AF itself can cause electrical as well as structural changes in atrial myocytes. Further studies have shown that longer episodes of AF can indeed also cause damage to the cellular structure of atrial myocytes including the contractile apparatus, cellular organelles or cause cellular death. These structural changes induced by AF itself are summarized as AF-induced atrial cardiomyopathy. Nevertheless, other cardiovascular diseases can cause substantial atrial alterations (atrial cardiomyopathies) without the presence of AF [3]. Thus, in many patients with AF the atrial cardiomyopathy appears as the driving factor for the development of AF in the future. Therefore, the first experimental studies about electrical remodeling do not resemble the full spectrum of electrophysiological and structural changes seen in patients with different types of AF [3,4,5,6,7,8].

## 2. Atrial Cardiomyopathy

Atrial cardiomyopathy is defined as any complex of structural, architectural, contractile or electrophysiological changes affecting the atria with the potential to produce clinically-relevant manifestations [3]. The term EHRAS (for European Heart Rhythm Association; EHRA/Heart Rhythm Society; HRS/Asian Pacific Heart Rhythm Association; APHRS/Latin American Society of Electrophysiology and Cardiac Stimulation; SOLAECE) classification was introduced by this consensus report. Four EHRAS classes were defined: (I) principal cardiomyocyte changes; (II) principally fibrotic changes; (III) combined cardiomyocyte-pathology/fibrosis; and (IV) primarily non-collagen infiltration (with or without cardiomyocyte changes). Nevertheless, EHRAS classes are not static and may vary over time, and therefore the EHRAS classification is purely descriptive [3]. cardiomyopathy is defined as any complex of structural, architectural, contractile or electrophysiological changes affecting the atria with the potential to produce clinically-relevant manifestations [3]. The term EHRAS (for EHRA/HRS/APHRS/SOLAECE) classification was introduced by this consensus report. Four EHRAS classes were defined: (I) principal cardiomyocyte changes; (II) principally fibrotic changes; (III) combined cardiomyocyte-pathology/fibrosis; and (IV) primarily non-collagen infiltration (with or without cardiomyocyte changes). Nevertheless, EHRAS classes are not static and may vary over time, and therefore, the EHRAS classification is purely descriptive [3].

## 3. Oxidative Stress as Central Mediator for Atrial Electrical and Structural Remodeling in AF

“Oxidative stress” has been implicated in the pathogenesis of multiple cardiac/cardiovascular disease conditions among them myocardial infarction, myocardial ischemia/reperfusion, heart failure, diabetic cardiomyopathy, and cardiotoxicity [9,10,11,12,13].

**Figure 1:**
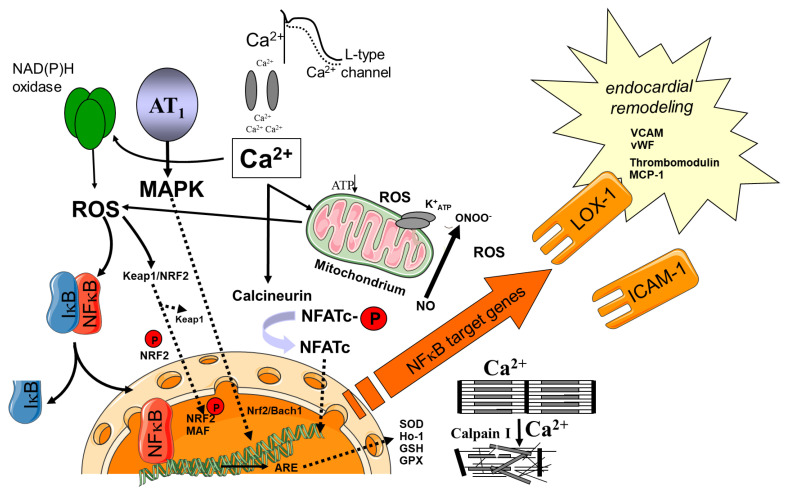
Association between atrial fibrillation (AF) with cellular calcium overload, shortening of the action potential, oxidative stress (reactive oxygen species; ROS) and activation/expression of several pathways and genes, which interfere with cellular morphology/function and atrial thrombogenesis. Angiotensin II can modify several effects through activation of the AT1 receptor. Activation of different Mitogen activated kinases (MAP kinases) induce several cellular effects including cellular hypertrophy. In addition, calcium-activated phosphatases (calcineurin) and protease (calpain I) induce structural cellular changes. Oxidative stress is counterbalanced to some extent by activation of the antioxidant response element (ARE) and generation of superoxide dismutase (SOD), glutathione (GSH), glutathione peroxidase (GPX). NRF2 = nuclear factor-erythroid-2-related factor, Keap1 = Kelch-like ECH-associated protein 1.

For decades, “oxidative stress” has taken center stage in its role as a main contributor to development and progression of AF and as a mediator of AF-induced atrial remodeling processes (Figure 1). A number of AF risk factors are closely associated with the increased formation of ROS. AF and a number of AF risk factors are closely related. Mechanistically, for example, obesity, diabetes and high blood pressure share their proarrhythmogenic effects via increased formation of ROS/RNS, which can aggravate inflammation and fibrosis. Thus, special emphasis is given here to the role of ROS/RNS in atrial cardiomyopathy.

Of note, reactive oxygen species (ROS) are formed to some extent under physiological conditions where they are indispensable in maintaining cell signaling, redox homeostasis, proliferation, differentiation, and cell viability [14]. ROS can selectively alter the redox status of various molecular targets which quite specifically leads to functional alterations of, e.g., ion channel activity or activation of a variety of redox-sensitive signal transduction pathways. It is an excessive ROS production, however, that has been associated with pathogenesis cardiovascular disease [14]. Different oxidants affect distinct sets of target proteins through modifications that are specific both with respect to the oxidant and the site of modification. H_2_O_2_, for instance, preferentially oxidizes the thiol side chain of well-defined cysteinyl residues in targeted functional motifs [15]. The antioxidant equipment considerably differs between cell types and conditions cells are exposed to with respect to quantity and specificity. Furthermore, the antioxidant redox systems in the different cellular compartments, e.g., glutathione, NADPH, thioredoxin (Trx), and peroxidases, are not in equilibrium and are independently maintained at distinct redox potentials. Accordingly, in a more contemporary way, “oxidative stress” may be defined as the chronic disturbance of redox circuits and redox-responsive signal transduction pathways [16,17,18].

AF is the most common arrhythmia in clinical practice and has been repeatedly associated with increased “oxidative stress” (Figure 1). Evidence has been accumulated pointing to a linkage of “oxidative stress” to atrial remodeling during AF [19,20,21]. For reasons not fully understood, the efficacy of antioxidants to prevent AF and AF-dependent tissue remodeling has been largely disappointing.

Mihm et al. [21] were the first to demonstrate extensive oxidative damage in atrial myocardium from patients with AF. The oxidative damage was mainly mediated by peroxynitrite and was not observed in samples from patients in sinus rhythm (SR). Later it was shown that rapid atrial pacing lowers tissue ascorbic acid levels, whereas the abundancy of nitrated proteins increased along with a shortening of effective refractory period (ERP) [22]. Pre-treatment with vitamin C largely abolished all of these changes, indicating that tachycardia does effectively increase the concentration of ROS and RNS. The resulting nitration and carbonylation of cellular proteins impair myocardial energetic and electrophysiologic properties. Similarly, rapid pacing of in vitro differentiated cardiomyocytes [23] or human atrial tissue slices provoked oxidative modifications of proteins and mitochondrial dysfunction [24].

It has been shown that the cardiac excitation-contraction coupling components are particularly sensitive to oxidative stress and, thus, contribute to AF substrate formation [22,25,26,27,28]. However, dysregulation of ion channel function and Ca^2+^-handling have been identified as key phenomena contributing already to the onset of AF [29]. Along this line, it has been shown recently that the increased AF-inducibility and AF-duration observed in mouse models of type 1 diabetes mellitus is due to down-regulation of SCN5a/NaV1.5 gene expression in the absence of insulin [30]. Mitochondrial antioxidant therapy abrogated these alterations and reversed the obesity-induced AF burden. Indeed, diabetes mellitus and obesity are established risk factors not only for AF, but also for the development of CAD and diabetic cardiomyopathy [31,32,33,34]. Similarly, inducible AF in obese mice was found to result from a combined sodium, potassium, and calcium channel remodeling and was accompanied by atrial fibrosis [35]. The receptor of advanced-glycation end products (AGE) and signaling and hyperglycemia-dependent oxidative stress are the main drivers of diabetes-induced cardiac remodeling, which comprises cardiac (atrial) fibrosis, impaired Ca^2+^-handling myocyte hypertrophy, and increased apoptosis [33]. Specifically, mitochondrial dysfunction and mitochondria-related oxidative stress have been detected in CHD patients and were found to be elevated in patients with CHD and DM [36]. AF itself has substantial effects on mitochondrial structure and function: Lin et al. [37] described oxidative damage of mitochondrial DNA and reduced ATP synthesis along with increased production of ROS that were due to initial calcium-overload. These findings could be confirmed by later studies [24]. A recent proteomics study identified mitochondrial dysfunction, oxidative phosphorylation, glutathione redox reaction I, Nrf2-mediated oxidative stress response, and hepatic fibrosis among the ten top regulated canonical pathways in AF [38].

Increased production of ROS/RNS occurs immediately after new-onset AF as has been shown in patients with lone recurrent AF and for rapid atrial pacing models [25,39,40,41]. Acute episodes of AF induce redox-sensitive signaling/gene expression in the LV myocardium and compromise microvascular blood flow [7,24,42].

Gene expression profiling of atrial tissue samples from patients with SR and AF revealed a decreased expression of anti-oxidative genes, whereas that of several ROS-producing genes was increased [40]. A recent large-scale transcriptome approach [43] and a meta-analysis of available transcriptome data [44] confirmed the ample AF-dependent ion channel remodeling as well as the involvement of the inter-related oxidative stress, inflammation, and fibrosis pathways.

Redox-regulation and mounting of anti-oxidative response mechanisms involves three important groups of enzymes, the glutaredoxins (GRX), peroxiredoxins (PRX), and thioredoxins (TRX; including the thioredoxin interacting protein, TXNIP). In the setting of AF, not much is known on their expression, activity, and specific function. Available data indicate decreased expression of PRX3 (along with decreased superoxide dismutase (SOD) levels) in atrial tissue of dogs subjected to two weeks of ventricular tachypacing [45]. In acute rapid pacing in vivo (pig), elevated atrial mRNA expression of thioredoxin reductase (TXNRD1), PRX3, and SOD2 has been observed [7,42]. Recently, a protective role against mitochondrial oxidative stress and diabetes-induced hypertrophy has been assigned to thioredoxin 2 [46].

## 4. Sources of ROS/RNS

Several mechanisms and sources contribute to elevated ROS/RNS levels in cardiovascular disease, and during AF in particular (Figure 1). Mitochondria represent one major source of ROS and, during AF in response to existing risk factors, show substantial structural and morphological alterations (e.g., swelling and loss of cristae structure), but also become compromised functionally [23,24,47,48]. This is associated with increased production of superoxide anion radicals by the respiratory chain [49], particularly at complexes I and III of the respiratory chain [50]. Mitochondrial morphology and function could be largely preserved by limiting calcium influx via blockage of L-type calcium channels with verapamil. Likewise, mibefradil, which blocks L-type and T-type Ca^2+^ prevented the oxidation of cellular constituents and showed cytoprotective effects [51].

Similar to AF, ischemia also provokes alterations in cellular ionic homeostasis, in particular of calcium and sodium ions. Thereby, ischemia creates a substrate for AF maintenance [52,53]. Increased NCX currents and spontaneous Ca^2+^-release events contribute to increased spontaneous ectopy [54]. In pulmonary veins (PV), hypoxia-induced EAD and DAD as well as reoxygenation-induced PV burst firing represent important pro-arrhythmogenic mechanisms [55].

Besides mitochondria, a family of seven NADPH-dependent enzymes consisting of the “NADPH oxidases” Nox 1–5 and Duox 1–2 represent a second important source of ROS during AF [56]. Different isoforms for the key catalytic subunit, Nox, exist in nonphagocytic cells, including cardiac myocytes, fibroblasts, and endothelial cells. Although non-phagocyte NADPH oxidases show some constitutive activity, their activity can be further up-regulated in response to a broad variety of stimuli that are common to major risk factors of AF such as hypertension and diabetes and include angiotensin II (AngII), endothelin-1 (ET-1), growth factors, cytokines, and mechanical stress [57,58,59,60]. Nox2 and Nox4 are the isoforms predominantly expressed in cardiac cells. Nox1 is expressed particularly in vascular smooth muscle cells and responsible for extracellular superoxide production in coronary arterial myocytes [61]. Nox4 produces mainly hydrogen peroxide and only small amounts of superoxide anion intracellularly [62]. In cardiomyocytes, increased Nox4 expression is associated with mitochondrial dysfunction and apoptosis [63,64]. Overexpression of Nox4 in zebrafish embryos induces an arrhythmogenic phenotype [65], an effect mediated by increased ROS production and subsequent activation of CaMKII. Nox1 contributes to the hypertensive response to AngII [61,66]. Increased left ventricular (LV) expression of Nox2, Nox1, and Nox4 has been observed in an animal model of acute AF [7,42]. The AT1 receptor antagonist, irbesartan, and the multichannel inhibitor, dronedarone, efficiently prevented the up-regulation of Nox2 [7,42]. Apocynin, an inhibitor of Nox, attenuated RAP-dependent electrical remodeling, AF inducibility and duration in rabbits [67]. Myocardial overexpression of Nox2 in mice elevated superoxide production in the atria and led to a moderate increase in AF-inducibility [68]. However, there was no indication of any electrical or structural atrial remodeling going on and, accordingly, it is concluded that Nox2 overexpression and resulting elevated amounts of superoxide do not contribute to the maintenance of AF.

A shift from NADPH oxidase to other cellular sources of ROS which include xanthine oxidase, [69], monoamine oxidase [70], and uncoupled eNOS [71] occurs with increasing duration of AF. This mechanism agrees with the observation that statins, which reduce ROS production by NADPH oxidases via inhibition of Rac1, are effective in acute models of AF and in patients with post-operative AF, but fail to do so in models of long-lasting AF or patients with permanent AF. Furthermore, xanthine oxidase inhibition prevented AF induction by preventing both electrical and structural remodeling [69].

eNOS is a homodimeric enzyme that oxidises l-arginine to NO and l-citrullin. For this reaction, tetrahydrobiopterin (BH4) and Ca^2+^-activated calmodulins are essential cofactors [72]. BH4 facilitates enzyme dimerization and stabilizes the active form that produces predominantly NO [73]. In the absence of BH4 or upon its oxidation, eNOS uncouples to monomers which produce large amounts of superoxide anions and, subsequently, of ONOO^−^ instead [72]. Reduced expression of eNOS further contributes to reduced plasma levels of NO observed during AF [74] which are restored after successful cardioversion into sinus rhythm [75,76].

Furthermore, AF is associated with increased levels of ADMA, an endogenous inhibitor of eNOS [77,78]. Upon restoration of sinus rhythm ADMA levels return to normal within 24h. AF and RAP per se up-regulate ADMA and may thereby cause the microcirculatory flow abnormalities observed in AF [77]. Altered NO generation is also known to influence mechanical performance of the ventricles and, therefore, increased ADMA levels might be related to abnormalities in Ca^2+^ handling and contractility [79,80]. AF or rapid pacing have been shown to reduce eNOS expression, an effect which could be attenuated by the angiotensin type 1 receptor blocker, olmesartan [81]. Similarly, losartan prevented the reduction of eNOS expression after myocardial infarction, which prevented subsequent AF induction [82]. However, alterations in eNOS expression and activity might depend on concomitant diseases, rather than on the presence of AF per se. This view is supported by an immunohistological study showing no independent association of AF and endocardial/myocardial eNOS expression in atrial samples [83].

In right atrial appendages (RAA), monoamine oxidase (MAO) has been identified as a substantial source of ROS [70]. MAO, a mitochondrial enzyme, catalyzes the oxidative deamination and, thus, inactivation of catecholamines. H_2_O_2_ is formed as a by-product in this reaction. MAO plays a causal role in cardiac dysfunction in response to pressure overload due to oxidative stress [84,85]. MAO is associated with an increased risk for post-operative-AF [70].

### 4.1. Redox-Regulated Signaling Pathways

Although the excessive production of ROS/RNS contributes to cellular damage, physiological levels rather induce reversible and site-specific protein modifications that define “redox signaling” processes which are subject to stringent spatial and temporal regulation [86]. Covalent modification of cysteine thiols or oxidation of iron-sulfur-clusters in proteins, S-glutathionylation, and S-nitrosylation/S-nitrosation (SNO) have all been identified as major mechanisms mediating ROS-specific effects (for review see [86]). It is well established that these mechanisms contribute to AF development and progression by altering e.g., ion channel activity. More recently, the oxidation of methionine which results in the formation of methionine sulfoxide, and the regeneration of methionine by the thioredoxin-dependent methionine sulfoxide reductase (Msr) has been identified as an additional mechanism linking ROS with AF [19,87]. In their conclusive study, Purohit et al. show that oxidized Ca^2+^ and calmodulin-dependent protein kinase II (CaMKII) plays a crucial role in mediating the AngII/RAP-dependent induction of AF [87]. AngII, elevated plasma and tissue levels of which are common to most risk factors for AF, and RAP were shown to turn CaMKII constitutively active by the oxidation of its methionine amino acid residues 281/282 [87,88]. This increase in the amounts of oxidized CaMKII occurred without any changes of total amounts of CaMKII. The substantial contribution of AngII in this activation has been emphasized by demonstrating that inhibitors of angiotensin-converting enzyme (ACEi) or angiotensin II type I receptor blockers (ATRBs) prevent CaMKII oxidation/activation [87]. Mechanistically, activated CaMKII increases Ca^2+^-leak from the sarcoplasmic reticulum via enhanced phosphorylation of RyR2 [89]. Elevated diastolic Ca^2+^ concentrations are responsible for the resulting increase in AF inducibility and duration. In this way, CaMKII functions as a cellular sensor for ROS that links the important pathogenetic factors AngII, Nox, and Ca^2+^ with AF.

Nuclear factor-κB (NF-κB) is a key transcription regulator coupling redox state to the transcriptional regulation in various pathophysiological settings, including AF [90]. Elevated intracellular calcium levels, AngII and ROS, PDGF, CTGF and TGF-β1, all associated with AF-dependent structural remodeling, are major activators of the immediate early response transcription factor NF-κB [7,17,24,91,92,93,94,95,96]. Interestingly, increased Rac-1 levels may also contribute to NF-κB activation during RAP or AF [97,98,99].

The typical target genes of NF-κB comprise pro-inflammatory cytokines such as interleukin-8 and TNFα, but also endothelial adhesion molecules ICAM-1 and VCAM-1, the cardiac sodium channel SCN5A, and the endothelial oxidized low-density lipoprotein (lectin-like) receptor, LOX-1 [24,90,91,92]. NF-κB contributes also to the induction of heme oxygenase 1 (HO-1) expression [100]. HO-1 is a redox-sensitive inducible protein that supports cytoprotection against “oxidative stress” under diverse unrelated conditions [101]. The HO-1 promoter contains responsive elements for NF-κB, AP-1 and 2, and Nrf2, the latter being of particular importance for HO-1 expression [102].

RAP and AF not only contribute to structural, electrical, and endocardial/endothelial remodeling in the atria; rapid atrial pacing has been shown to activate the NF-κB pathway also in the left ventricle [7]. In line with this ventricular activation of NF-κB, established target genes of NF-κB were up-regulated which included VEGFA [103,104], Fn14, CCL2 [105], HIF1A [95,106] as well as DnaJ family members. DNAJA4 and DNAJB9 are co-chaperones for the ATPase activity of Hsp70 and protect stressed cells from apoptosis [107]. DNAJA4 and DNAJB9 are both antioxidant response element (AREs) -regulated genes which become activated through nuclear factor-erythroid 2-related factor 2 (Nrf2) in response to oxidative stress. After phosphorylation by e.g., PKC, Nrf2 translocates to the nucleus where it binds to AREs and trans-activates target genes including enzymes such as peroxiredoxin I that regulate the intracellular amounts of ROS [108].

The Nrf2 signaling pathway is closely linked to the development of cardiac diseases such as AF, diabetic cardiomyopathy, myocarditis, heart failure, and ischemic heart disease [109]. Recent data indicate that the cellular redox state is subject to regulation by miRNAs through modulating Nrf2-dependent anti-oxidant gene expression or attenuating activities of ROS handling enzymes [110]. These mechanisms in turn can affect miRNA expression/activity [111,112,113]. Mounting evidence demonstrates the importance of miRNAs for the regulation of cardiac physiology [114]. In particular, miRNAs regulate cardiac excitability and arrhythmogenesis, as first shown for the abundantly expressed miRNAs in the heart, miR-1, -133, and -328 [115,116,117]. Meanwhile, the number of miRNAs with an established role in cardiac remodeling or altered expression during AF has substantially increased [114,118,119,120]. Of these AF-associated miRNAs, a few have been already identified as regulators of ROS generation (miR-25, miR-146a) [110].

Hypoxia-inducible factors (Hifs) are heterodimeric factors that control a hypoxia-induced gene expression profile aimed at counteracting adverse cellular and systemic effects of limitations in oxygen supply. Of the three mammalian isoforms, Hif1α probably is the most intensive studied one. Hif1α levels are regulated by oxygen tension at transcriptional, translational, and post-translational level, with O_2_-dependent protein stability being the best characterized regulatory mechanism [121]. Under normoxic conditions, the sufficiently available O_2_ is used by prolyl-hydroxylase domain-containing enzymes (PHD) for the corresponding modification of Hif1α, which then becomes a target for ubiquitinoylation by von Hippel-Lindau complex and proteasomal degradation [121]. Hypoxia prevents this degradation and, thereby enables the binding of Hif1α/β heterodimers to hypoxia-response elements in the promoters of target genes. These include metabolic and angiogenic factors. In response to RAP or during AF, increased Hif-1α expression has been observed, together with elevated expression of the Hif-target genes, VEGFA and PPARGC1α [42]. Both factors are induced in response to hypoxia or deprivation of nutrients [122,123]. Under the same conditions, and independent of this canonical HIF-pathway, elevated PPARGC1α levels exert strong angiogenic activity and induce VEGF expression by co-activating ERR-α [122]. Thus, both HIF-1α and PPARGC1α mediate the angiogenic response to AF-dependent flow alterations and may provide protection against ischemic damage.

Lysyl oxidase (LOX) is another established target gene of HIF. Accordingly, LOX expression was found to be induced in response to hypoxia/ischemia. Recent work established a role of LOX in cardiovascular function and disease, including AF [124,125,126,127]. LOX is a copper-dependent amine oxidase expressed and secreted by vascular smooth muscle cells and other fibrogenic cells, which initiates the cross-linking of collagen and elastin [128]. During the oxidative deamination of (hydroxyl)-lysine residues highly reactive semi-aldehydes and H_2_O_2_ are generated. Catalytic forms of LOX have been also detected in nuclei and cytosol, but their function remains to be elucidated fully [129,130]. Increased expression of LOX together with increased collagen cross-linking has been observed in left atria of patients with AF [124]. AngII and Rac1 were shown to mediate the up-regulation of LOX expression [124]. If and to what extent H_2_O_2_ generated by LOX contributes to atrial redox signaling and the ROS-dependent generation of AF substrate is not clear, yet (Figure 1). However, LOX and LOX-derived H_2_O_2_ are potent chemoattractants for monocytes and VSMC [131,132], which could well contribute to atrial cardiomyopathy and AF-related remodeling [133,134].

### 4.2. Role of Cellular Inflammatory Pathways

**Figure 2:**
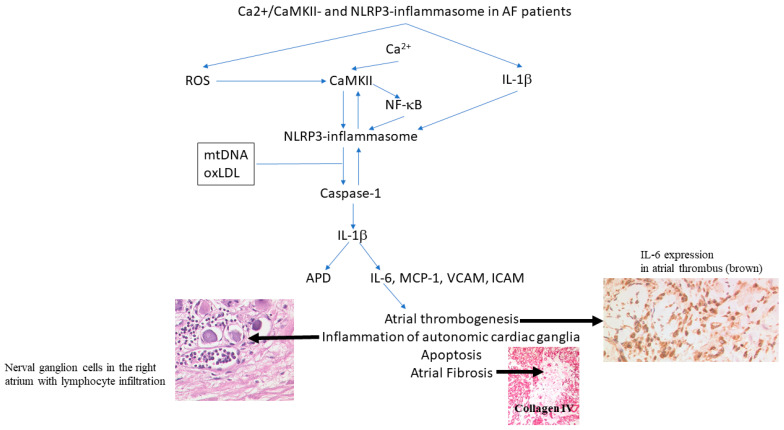
Interaction of atrial fibrillation (AF), CaMKII (Ca^2+^/calmodulin-dependent protein kinase-II), and NLRP3 (NACHT, LRR, and PYD domains-containing protein 3)-inflammasome.

The pathogenesis of several cardiovascular diseases including myocarditis, heart failure and myocardial infarction involves inflammation and inflammatory cytokines [135]. This has also demonstrated for AF [29]. Inflammation mediates structural remodeling, and fibrosis in particular, induces cellular damage, apoptosis, fibrosis, and subsequent (atrial) dilatation [136]. Inflammatory biomarkers associated with AF include interleukins-6, -8, and -2, CRP, TNFα, MCP-1, and HSP27 [137]. In atrial biopsy specimens from patients with lone AF, inflammatory infiltrates, myocyte necrosis, and fibrosis could be detected that were absent in the ventricles [138]. Mechanistically, it has been shown for TNFα, that it disturbs calcium homeostasis and activates the NPL3 inflammasome, thereby further aggravating inflammatory cytokine production (Figure 2) [139,140,141]. Of note, angiotensin II represents one major contributing factor in the induction of cardiac inflammation, which is partly mediated by activated c-Jun *N*-terminal kinase (JNK) and induction of TNFα [142,143]. CRP and interleukin-6 were shown to be positively correlated to left atrial diameter [144] and elevated levels increase the risk for vascular death and thromboembolic events [145]. CRP levels decrease after restoring sinus rhythm and predict the recurrence of AF [146,147,148,149].

### 4.3. Role of Adipose Tissue/Obesity and Diabetes

Obesity and metabolic syndrome, including diabetes, represent major risk factors for AF [150,151,152,153,154]. As mentioned above, AF and its risk factors are closely interwoven and, as a common mediator, share increased ROS production. With respect to adipose tissue, the risk for new-onset AF increases with each unit increment of the body mass index (BMI) by up to 8% [150,152]. In the general population, obesity leads to a 49% increased risk for AF [151]. Total visceral, epicardial, and intrathoracic fat provoke different effects on the cardiovascular system [155,156]. The epicardial adipose tissue (EAT) volume is closely associated with paroxysmal and persistent AF, independently of “classical” risk factors such as left atrial diameter (LAD) [157]. Amounts of EAT are independently related to AF recurrence after ablation [158]. Fibrosis represents a hallmark of structural atrial remodeling during AF [159] but, whenever induced in the heart, it serves as a powerful substrate for AF. Fibrosis can be induced by exogeneous factors [160], cardiovascular disease, and, according to the pioneering work of Hatem and his group, epicardial adipose tissue under certain clinical conditions is capable of inducing atrial fibrosis via adipokine secretion [161]. Furthermore, subepicardial adipose tissue could be replaced eventually by fibrosis [162]. Very recently, a key role in this process could be assigned to two distinct progenitor cell populations which become mobilized in the epicardial tissue under pathological conditions [163].

Comorbidities induce changes in the mitochondrial DNA (mtDNA) and oxLDL, which activates the atrial NLRP3 inflammasome. Reactive oxygen species (ROS) stimulate the NLRP3 inflammasome, which causes stimulation of caspase-1 maturates interleukin (IL)-1β. Thereby, IL-6 and CRP (C-reactive protein) are increased. IL-1β amplifies the NLRP3 inflammatory signaling. NF-κB, nuclear factor kappa-light-chain-enhancer of activated B cells (modified from [164,165]).

Total and LA EAT volumes as well as left periatrial EAT thickness are greater in persistent AF versus paroxysmal or no AF [158,166,167,168]. EAT distribution around the LA is uneven with most EAT located within regions superior vena cava, right pulmonary artery, right-sided roof of the LA, aortic root, pulmonary trunk, left atrial appendage, and between the left inferior PV and left atrioventricular groove [158,169]. EAT locations were associated with high dominant frequency (DF) sites, and thereby EAT is contributing to AF maintenance [167,170]. Recent data suggests that the contribution of EAT to the AF substrate may differ between LA and RA [170]. By releasing e.g., adipokines and pro-inflammatory cytokines, pericardial adipose tissue links AF with inflammation and obesity [168,171,172,173,174]. Elevated levels of CRP, interleukin-6 (IL-6), IL-8, tumor necrosis factor 1α (TNF1α), and of the adipokine, resistin, have been associated with AF [175]. Elevated post-operative serum levels of resistin seem to increase the risk of AF after coronary artery bypass graft surgery [176]. Expansion of adipose tissue leads to a chronic inflammatory response, mediated to a large extent via activation of JNK and NF-κB signaling [177]. Activated pro-inflammatory cytokine expression as well as metabolic changes contribute to the development of AF comorbidities [177]. In addition to these humoral effects, epicardial adipocytes can modulate the electrophysiological properties of neighboring cardiomyocytes by direct interaction [178]. Adipocyte accumulation within the myocardium may disturb atrial conduction and favor the development and persistence of re-entry circuits [179]. Likewise, the presence of intramyocardial adipose or lipomatous metaplasia increased the propensity to ventricular tachycardia in an ovine model of ischemic cardiomyopathy [180]. Both mechanisms may be supported by AF-dependent changes in atrial (or cardiac) gene expression. In addition to these humoral effects, epicardial adipocytes can modulate the electrophysiological properties of neighboring cardiomyocytes by direct interaction [177]. Adipocyte accumulation within the myocardium may disturb atrial conduction and favor the development and persistence of re-entry circuits [178]. Likewise, the presence of intramyocardial adipose or lipomatous metaplasia increased the propensity to ventricular tachycardia in an ovine model of ischemic cardiomyopathy [179]. Both mechanisms may be supported by AF-dependent changes in atrial (or cardiac) gene expression.

According to the meta-analysis presented by Huxley et al. [181], diabetic patients have a 40% higher risk of developing AF than non-diabetics. Oxidative stress and the resulting inflammation are established key mediators of AF in the diabetic, metabolically stressed heart [182,183,184,185]. Impaired electron transport through the respiratory chain and hyperglycemia have been identified as sources of elevated ROS production in animal models and diabetic patients [186,187]. Mechanistically, delayed after depolarizations (DADs) cause triggered activity to induce AF. DADs may result from calcium leakage from the sarcoplasmic reticulum via oxidation of ryanodine receptor 2 (RYR2). Calmodulin-dependent protein kinase II (CAMKII), normally activated by increased Ca^2+^ levels, could be alternatively activated by oxidation (e.g., by mitochondrial ROS) [184,188]. Advanced glycation end-products (AGEs) are other essential mediators between diabetes, overweight, and AF. AGEs represent an inhomogeneous group of lipids and proteins which are formed by non-enzymatic glycation and, therefore, their formation rate correlates with the average blood sugar level. Under diabetic conditions, but also in obesity and metabolic dysfunction, AGEs accumulate and increase cardiac stiffness by crosslinking matricellular and extracellular matrix proteins and trigger pro-inflammatory signaling via binding to receptors of AGE (RAGE), preferentially on fibroblasts [189]. In addition to cytokine induction, RAGE activation stimulates fibroblast proliferation and extracellular matrix production [190]. This pro-fibrotic process is substantially mediated by transforming growth factor-β (TGF-β) [191]. The activation of the local renin-angiotensin-system and in particular its classical angiotensin II (AngII)—angiotensin II type I receptor (AT1R) axis represents a hallmark of AF [192,193,194,195]. Similar to other pathogenetic factors, also increased local and systemic AngII levels have been associated with all four determinants of the metabolic syndrome, namely hypertension, hyperglycemia, obesity, and hyperlipidemia [196,197,198]. AngII affects the differentiation of progenitor cells and pre-adipocytes [199,200,201,202]. Accordingly, AT1R blockers and ACE inhibitors were shown to influence adipogenesis and adipocyte function, including the production and release of adipokines [203,204,205,206,207,208].

## 5. Atrial Endocardial Remodeling as a Cause of Thrombogenesis and Stroke

**Figure 3:**
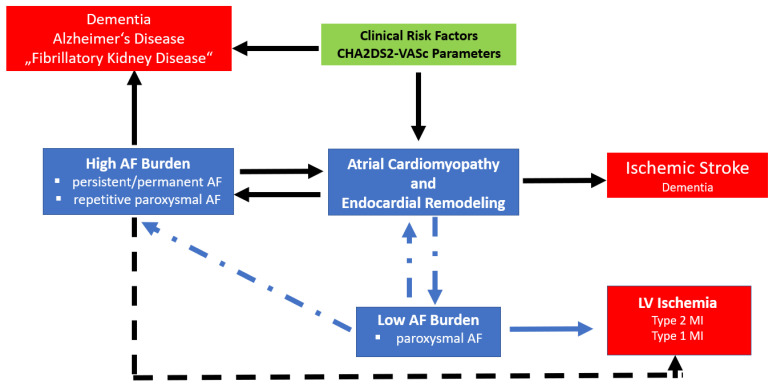
Impact of type of atrial fibrillation (paroxysmal AF, persistent AF) on clinical outcome. The recent hypothesis is that the burden of AF contributes to differences in the occurrence of specific clinical events such as cognitive decline, stroke, or myocardial infarction. At the molecular level, differences can be explained by activation of a difference in cellular oxidative stress pathways, which are regulated or counter-regulated during the course of an AF episode. Impact of type of atrial fibrillation (paroxysmal AF, persistent AF) on clinical outcome. The recent hypothesis is that the burden AF contributes to differences in the occurrence of specific clinical events such as cognitive decline, stroke or myocardial infarction. At the molecular level, differences can be explained by activation of difference cellular oxidative stress pathways, which are regulated or counter-regulated during the course of an AF episode. Diseases or conditions such as diabetes mellitus, history of stroke, heart failure, ageing, sex, and vascular diseases are summarized in the CHA2DS2VASc Score. Impact of type of atrial fibrillation (paroxysmal AF, persistent AF) on clinical outcome. The recent hypothesis is that the burden of AF contributes to differences in the occurrence of specific clinical events such as cognitive decline, stroke or myocardial infarction. At the molecular level, differences can be explained by activation of differences in cellular oxidative stress pathways, which are regulated or counter-regulated during the course of an AF episode.

The concept of ‘endocardial remodelling’ in AF was introduced by the consensus document on atrial cardiomyopathies [3]. In accordance with Virchow’s triad hypercoagulability, flow abnormalities, and endothelial changes co-exist to cause atrial thrombogenesis (Figure 1). Studies have demonstrated endocardial changes in atrial tissue specimens. Prothrombogenic factors (vWF, adhesion molecules VCAM-1, P-selectin, MCP-1) are expressed at the surface of endothelial cells causing platelets and leucocytes to adhere to the atrial endocardium in particular in the left atrial appendage [83,193,209], which causes the initiation of thrombus formation [209,210]. Diseases or conditions such as diabetes mellitus, heart failure, and ageing (CHA2DS2VASc Parameters) are known to increase alterations by oxidative stress pathways within endothelial cells, and thereby further increase the expression of prothrombogenic molecules. These changes are not related to the presence of absence of AF, explaining why atrial thrombogenesis is still increased even during sinus rhythm in certain subgroups of patients [211,212]. Recently, post hoc studies from large randomized controlled trials suggested that the burden of AF (Figure 3) as well as the type of AF (paroxysmal versus persistent/permanent AF) might be a factor, which drives differences in patient outcome [213,214,215,216,217]. Such clinical differences might be explained at least to some extent by differences in atrial cardiomyopathies and ventricular oxidative stress levels during various types of AF [210].

## 6. Conclusions

ROS/RNS and redox signaling have been implicated as the central component in the process of atrial remodeling. Redox-signaling contributes to tissue remodeling and self-perpetuation of AF. Nevertheless, so far antioxidant/redox-modifying therapies have not been shown to prevent, terminate, or reverse AF-induced atrial remodeling.

## Data Availability

Not applicable.

## References

[1] Wijffels M.C., Kirchhof C.J., Dorland R., Allessie M.A. (1995). Atrial fibrillation begets atrial fibrillation. A study in awake chronically instrumented goats. Circulation.

[2] Goette A., Honeycutt C., Langberg J.J. (1996). Electrical remodeling in atrial fibrillation. Time course and mechanisms. Circulation.

[3] Goette A., Kalman J.M., Aguinaga L., Akar J., Cabrera J.A., Chen S.A., Chugh S.S., Corradi D., D’Avila A., Dobrev D. (2016). EHRA/HRS/APHRS/SOLAECE expert consensus on atrial cardiomyopathies: Definition, characterization, and clinical implication. EP Eur..

[4] Bukowska A., Felgendreher M., Scholz B., Wolke C., Schulte J.S., Fehrmann E., Wardelmann E., Seidl M.D., Lendeckel U., Himmler K. (2018). CREM-transgene mice: An animal model of atrial fibrillation and thrombogenesis. Thromb. Res..

[5] Bukowska A., Spiller L., Wolke C., Lendeckel U., Weinert S., Hoffmann J., Bornfleth P., Kutschka I., Gardemann A., Isermann B. (2017). Protective regulation of the ACE2/ACE gene expression by estrogen in human atrial tissue from elderly men. Exp. Biol. Med..

[6] Chilukoti R.K., Mostertz J., Bukowska A., Aderkast C., Felix S.B., Busch M., Volker U., Goette A., Wolke C., Homuth G. (2013). Effects of irbesartan on gene expression revealed by transcriptome analysis of left atrial tissue in a porcine model of acute rapid pacing in vivo. Int. J. Cardiol..

[7] Goette A., Bukowska A., Dobrev D., Pfeiffenberger J., Morawietz H., Strugala D., Wiswedel I., Rohl F.W., Wolke C., Bergmann S. (2009). Acute atrial tachyarrhythmia induces angiotensin II type 1 receptor-mediated oxidative stress and microvascular flow abnormalities in the ventricles. Eur. Heart J..

[8] Torp-Pedersen C., Goette A., Nielsen P.B., Potpara T., Fauchier L., John Camm A., Arbelo E., Boriani G., Skjoeth F., Rumsfeld J. (2020). ‘Real-world’ observational studies in arrhythmia research: Data sources, methodology, and interpretation. A position document from European Heart Rhythm Association (EHRA), endorsed by Heart Rhythm Society (HRS), Asia-Pacific HRS (APHRS), and Latin America HRS (LAHRS). EP Eur..

[9] Shah A.K., Bhullar S.K., Elimban V., Dhalla N.S. (2021). Oxidative Stress as A Mechanism for Functional Alterations in Cardiac Hypertrophy and Heart Failure. Antioxidants.

[10] Singal P.K., Khaper N., Farahmand F., Bello-Klein A. (2000). Oxidative stress in congestive heart failure. Curr. Cardiol. Rep..

[11] Singal P.K., Khaper N., Palace V., Kumar D. (1998). The role of oxidative stress in the genesis of heart disease. Cardiovasc. Res..

[12] Takimoto E., Kass D.A. (2007). Role of oxidative stress in cardiac hypertrophy and remodeling. Hypertension.

[13] Cai L., Kang Y.J. (2001). Oxidative stress and diabetic cardiomyopathy: A brief review. Cardiovasc. Toxicol..

[14] Liang J., Wu M., Chen C., Mai M., Huang J., Zhu P. (2020). Roles of Reactive Oxygen Species in Cardiac Differentiation, Reprogramming, and Regenerative Therapies. Oxidative Med. Cell. Longev..

[15] Rhee S.G. (2006). Cell signaling. H_2_O_2_, a necessary evil for cell signaling. Science.

[16] Ghezzi P., Bonetto V., Fratelli M. (2005). Thiol-disulfide balance: From the concept of oxidative stress to that of redox regulation. Antioxid Redox Signal..

[17] Goette A., Bukowska A., Lillig C.H., Lendeckel U. (2012). Oxidative Stress and Microcirculatory Flow Abnormalities in the Ventricles during Atrial Fibrillation. Front. Physiol..

[18] Jones D.P. (2006). Redefining oxidative stress. Antioxid. Redox Signal..

[19] Yang K.C., Dudley S.C. (2013). Oxidative stress and atrial fibrillation: Finding a missing piece to the puzzle. Circulation.

[20] Negi S., Sovari A.A., Dudley S.C. (2010). Atrial fibrillation: The emerging role of inflammation and oxidative stress. Cardiovasc. Hematol. Disord. Drug Targets.

[21] Mihm M.J., Yu F., Carnes C.A., Reiser P.J., McCarthy P.M., Van Wagoner D.R., Bauer J.A. (2001). Impaired myofibrillar energetics and oxidative injury during human atrial fibrillation. Circulation.

[22] Carnes C.A., Chung M.K., Nakayama T., Nakayama H., Baliga R.S., Piao S., Kanderian A., Pavia S., Hamlin R.L., McCarthy P.M. (2001). Ascorbate attenuates atrial pacing-induced peroxynitrite formation and electrical remodeling and decreases the incidence of postoperative atrial fibrillation. Circ. Res..

[23] Schild L., Bukowska A., Gardemann A., Polczyk P., Keilhoff G., Tager M., Dudley S.C., Klein H.U., Goette A., Lendeckel U. (2006). Rapid pacing of embryoid bodies impairs mitochondrial ATP synthesis by a calcium-dependent mechanism—A model of in vitro differentiated cardiomyocytes to study molecular effects of tachycardia. Biochim. Biophys. Acta.

[24] Bukowska A., Schild L., Keilhoff G., Hirte D., Neumann M., Gardemann A., Neumann K.H., Rohl F.W., Huth C., Goette A. (2008). Mitochondrial dysfunction and redox signaling in atrial tachyarrhythmia. Exp. Biol. Med..

[25] Ben Abraham R., Matza M., Marmor S., Rudick V., Frolkis I., Shapira I., Weinbroum A.A. (2003). Electromechanical impairment of human auricle and rat myocardial strip subjected to exogenous oxidative stress. Eur. J. Cardio-Thorac. Surg. Off. J. Eur. Assoc. Cardio-Thorac. Surg..

[26] Dhalla N.S., Temsah R.M. (2001). Sarcoplasmic reticulum and cardiac oxidative stress: An emerging target for heart disease. Expert Opin. Ther. Targets.

[27] Dhalla N.S., Temsah R.M., Netticadan T. (2000). Role of oxidative stress in cardiovascular diseases. J. Hypertens..

[28] Loh S.H., Jin J.S., Tsai C.S., Chao C.M., Tsai Y., Chen W.H., Cheng T.H., Chuang C.C., Lin C.I. (2003). Possible underlying mechanism for hydrogen peroxide-induced electromechanical suppression in human atrial myocardium. J. Pharmacol. Sci..

[29] Andrade J., Khairy P., Dobrev D., Nattel S. (2014). The clinical profile and pathophysiology of atrial fibrillation: Relationships among clinical features, epidemiology, and mechanisms. Circ. Res..

[30] Polina I., Jansen H.J., Li T., Moghtadaei M., Bohne L.J., Liu Y., Krishnaswamy P., Egom E.E., Belke D.D., Rafferty S.A. (2020). Loss of insulin signaling may contribute to atrial fibrillation and atrial electrical remodeling in type 1 diabetes. Proc. Natl. Acad. Sci. USA.

[31] Boudina S., Abel E.D. (2007). Diabetic cardiomyopathy revisited. Circulation.

[32] Go A.S., Mozaffarian D., Roger V.L., Benjamin E.J., Berry J.D., Borden W.B., Bravata D.M., Dai S., Ford E.S., Fox C.S. (2013). Heart disease and stroke statistics—2013 update: A report from the American Heart Association. Circulation.

[33] Rosa C.M., Xavier N.P., Henrique Campos D., Fernandes A.A., Cezar M.D., Martinez P.F., Cicogna A.C., Gimenes C., Gimenes R., Okoshi M.P. (2013). Diabetes mellitus activates fetal gene program and intensifies cardiac remodeling and oxidative stress in aged spontaneously hypertensive rats. Cardiovasc. Diabetol..

[34] De Sensi F., Costantino S., Limbruno U., Paneni F. (2019). Atrial fibrillation in the cardiometabolic patient. Minerva Med..

[35] McCauley M.D., Hong L., Sridhar A., Menon A., Perike S., Zhang M., da Silva I.B., Yan J., Bonini M.G., Ai X. (2020). Ion Channel and Structural Remodeling in Obesity-Mediated Atrial Fibrillation. Circulation. Arrhythmia Electrophysiol..

[36] Duicu O.M., Lighezan R., Sturza A., Balica R., Vaduva A., Feier H., Gaspar M., Ionac A., Noveanu L., Borza C. (2016). Assessment of Mitochondrial Dysfunction and Monoamine Oxidase Contribution to Oxidative Stress in Human Diabetic Hearts. Oxidative Med. Cell. Longev..

[37] Lin P.H., Lee S.H., Su C.P., Wei Y.H. (2003). Oxidative damage to mitochondrial DNA in atrial muscle of patients with atrial fibrillation. Free. Radic. Biol. Med..

[38] Rennison J.H., Li L., Lin C.R., Lovano B.S., Castel L., Wass S.Y., Cantlay C.C., McHale M., Gillinov A.M., Mehra R. (2021). Atrial fibrillation rhythm is associated with marked changes in metabolic and myofibrillar protein expression in left atrial appendage. Pflügers Arch. Eur. J. Physiol..

[39] Ausma J., Wijffels M., Thone F., Wouters L., Allessie M., Borgers M. (1997). Structural changes of atrial myocardium due to sustained atrial fibrillation in the goat. Circulation.

[40] Kim Y.H., Lim D.S., Lee J.H., Shim W.J., Ro Y.M., Park G.H., Becker K.G., Cho-Chung Y.S., Kim M.K. (2003). Gene expression profiling of oxidative stress on atrial fibrillation in humans. Exp. Mol. Med..

[41] Loh S.H., Tsai Y.T., Lee C.Y., Chang C.Y., Tsai C.S., Cheng T.H., Lin C.I. (2014). Antiarrhythmic effects of dehydroevodiamine in isolated human myocardium and cardiomyocytes. J. Ethnopharmacol..

[42] Bukowska A., Hammwohner M., Sixdorf A., Schild L., Wiswedel I., Rohl F.W., Wolke C., Lendeckel U., Aderkast C., Bochmann S. (2012). Dronedarone prevents microcirculatory abnormalities in the left ventricle during atrial tachypacing in pigs. Br. J. Pharmacol..

[43] Steenman M. (2020). Insight into atrial fibrillation through analysis of the coding transcriptome in humans. Biophys. Rev..

[44] Haas Bueno R., Recamonde-Mendoza M. (2020). Meta-analysis of Transcriptomic Data Reveals Pathophysiological Modules Involved with Atrial Fibrillation. Mol. Diagn. Ther..

[45] De Souza A.I., Cardin S., Wait R., Chung Y.L., Vijayakumar M., Maguy A., Camm A.J., Nattel S. (2010). Proteomic and metabolomic analysis of atrial profibrillatory remodeling in congestive heart failure. J. Mol. Cell. Cardiol..

[46] Li H., Xu C., Li Q., Gao X., Sugano E., Tomita H., Yang L., Shi S. (2017). Thioredoxin 2 Offers Protection against Mitochondrial Oxidative Stress in H9c2 Cells and against Myocardial Hypertrophy Induced by Hyperglycemia. Int. J. Mol. Sci..

[47] Halestrap A.P. (2006). Calcium, mitochondria and reperfusion injury: A pore way to die. Biochem. Soc. Trans..

[48] Ravens U., Liu G.S., Vandeplassche G., Borgers M. (1992). Protection of human, rat, and guinea-pig atrial muscle by mioflazine, lidoflazine, and verapamil against the destructive effects of high concentrations of Ca^2+^. Cardiovasc. Drugs Ther. Spons. Int. Soc. Cardiovasc. Pharmacother..

[49] Cadenas E., Davies K.J. (2000). Mitochondrial free radical generation, oxidative stress, and aging. Free. Radic. Biol. Med..

[50] Schonfeld P., Wieckowski M.R., Lebiedzinska M., Wojtczak L. (2010). Mitochondrial fatty acid oxidation and oxidative stress: Lack of reverse electron transfer-associated production of reactive oxygen species. Biochim. Biophys. Acta.

[51] Mason R.P., Mak I.T., Walter M.F., Tulenko T.N., Mason P.E. (1998). Antioxidant and cytoprotective activities of the calcium channel blocker mibefradil. Biochem. Pharmacol..

[52] Rivard L., Sinno H., Shiroshita-Takeshita A., Schram G., Leung T.K., Nattel S. (2007). The pharmacological response of ischemia-related atrial fibrillation in dogs: Evidence for substrate-specific efficacy. Cardiovasc. Res..

[53] Sinno H., Derakhchan K., Libersan D., Merhi Y., Leung T.K., Nattel S. (2003). Atrial ischemia promotes atrial fibrillation in dogs. Circulation.

[54] Nishida K., Qi X.Y., Wakili R., Comtois P., Chartier D., Harada M., Iwasaki Y.K., Romeo P., Maguy A., Dobrev D. (2011). Mechanisms of atrial tachyarrhythmias associated with coronary artery occlusion in a chronic canine model. Circulation.

[55] Lin Y.K., Lai M.S., Chen Y.C., Cheng C.C., Huang J.H., Chen S.A., Chen Y.J., Lin C.I. (2012). Hypoxia and reoxygenation modulate the arrhythmogenic activity of the pulmonary vein and atrium. Clin. Sci..

[56] Afanas’ev I. (2011). ROS and RNS signaling in heart disorders: Could antioxidant treatment be successful?. Oxidative Med. Cell. Longev..

[57] Griendling K.K., Sorescu D., Ushio-Fukai M. (2000). NAD(P)H oxidase: Role in cardiovascular biology and disease. Circ. Res..

[58] Jaimes E.A., Galceran J.M., Raij L. (1998). Angiotensin II induces superoxide anion production by mesangial cells. Kidney Int..

[59] Yasunari K., Maeda K., Nakamura M., Yoshikawa J. (2002). Pressure promotes angiotensin II--mediated migration of human coronary smooth muscle cells through increase in oxidative stress. Hypertension.

[60] Li J.M., Gall N.P., Grieve D.J., Chen M., Shah A.M. (2002). Activation of NADPH oxidase during progression of cardiac hypertrophy to failure. Hypertension.

[61] Matsuno K., Yamada H., Iwata K., Jin D., Katsuyama M., Matsuki M., Takai S., Yamanishi K., Miyazaki M., Matsubara H. (2005). Nox1 is involved in angiotensin II-mediated hypertension: A study in Nox1-deficient mice. Circulation.

[62] Serrander L., Cartier L., Bedard K., Banfi B., Lardy B., Plastre O., Sienkiewicz A., Forro L., Schlegel W., Krause K.H. (2007). NOX4 activity is determined by mRNA levels and reveals a unique pattern of ROS generation. Biochem. J..

[63] Ago T., Kuroda J., Pain J., Fu C., Li H., Sadoshima J. (2010). Upregulation of Nox4 by hypertrophic stimuli promotes apoptosis and mitochondrial dysfunction in cardiac myocytes. Circ. Res..

[64] Kuroda J., Ago T., Matsushima S., Zhai P., Schneider M.D., Sadoshima J. (2010). NADPH oxidase 4 (Nox4) is a major source of oxidative stress in the failing heart. Proc. Natl. Acad. Sci. USA.

[65] Zhang Y., Shimizu H., Siu K.L., Mahajan A., Chen J.N., Cai H. (2014). NADPH Oxidase 4 Induces Cardiac Arrhythmic Phenotype in Zebrafish. J. Biol. Chem..

[66] Dikalova A., Clempus R., Lassegue B., Cheng G., McCoy J., Dikalov S., San Martin A., Lyle A., Weber D.S., Weiss D. (2005). Nox1 overexpression potentiates angiotensin II-induced hypertension and vascular smooth muscle hypertrophy in transgenic mice. Circulation.

[67] Zhang D.X., Ren K., Guan Y., Wang Y.T., Shan Z.L. (2014). Protective effects of apocynin on atrial electrical remodeling and oxidative stress in a rabbit rapid atrial pacing model. Chin. J. Physiol..

[68] Mighiu A.S., Recalde A., Ziberna K., Carnicer R., Tomek J., Bub G., Brewer A.C., Verheule S., Shah A.M., Simon J.N. (2021). Inducibility, but not stability, of atrial fibrillation is increased by NOX2 overexpression in mice. Cardiovasc. Res..

[69] Sakabe M., Fujiki A., Sakamoto T., Nakatani Y., Mizumaki K., Inoue H. (2012). Xanthine oxidase inhibition prevents atrial fibrillation in a canine model of atrial pacing-induced left ventricular dysfunction. J. Cardiovasc. Electrophysiol..

[70] Anderson E.J., Efird J.T., Davies S.W., O’Neal W.T., Darden T.M., Thayne K.A., Katunga L.A., Kindell L.C., Ferguson T.B., Anderson C.A. (2014). Monoamine oxidase is a major determinant of redox balance in human atrial myocardium and is associated with postoperative atrial fibrillation. J. Am. Heart Assoc..

[71] Reilly S.N., Jayaram R., Nahar K., Antoniades C., Verheule S., Channon K.M., Alp N.J., Schotten U., Casadei B. (2011). Atrial sources of reactive oxygen species vary with the duration and substrate of atrial fibrillation: Implications for the antiarrhythmic effect of statins. Circulation.

[72] Gielis J.F., Lin J.Y., Wingler K., Van Schil P.E., Schmidt H.H., Moens A.L. (2011). Pathogenetic role of eNOS uncoupling in cardiopulmonary disorders. Free. Radic. Biol. Med..

[73] Reif A., Frohlich L.G., Kotsonis P., Frey A., Bommel H.M., Wink D.A., Pfleiderer W., Schmidt H.H. (1999). Tetrahydrobiopterin inhibits monomerization and is consumed during catalysis in neuronal NO synthase. J. Biol. Chem..

[74] Cai H., Li Z., Goette A., Mera F., Honeycutt C., Feterik K., Wilcox J.N., Dudley S.C., Harrison D.G., Langberg J.J. (2002). Downregulation of endocardial nitric oxide synthase expression and nitric oxide production in atrial fibrillation: Potential mechanisms for atrial thrombosis and stroke. Circulation.

[75] Minamino T., Kitakaze M., Sato H., Asanuma H., Funaya H., Koretsune Y., Hori M. (1997). Plasma levels of nitrite/nitrate and platelet cGMP levels are decreased in patients with atrial fibrillation. Arterioscler. Thromb. Vasc. Biol..

[76] Nikitovic D., Zacharis E.A., Manios E.G., Malliaraki N.E., Kanoupakis E.M., Sfiridaki K.I., Skalidis E.I., Margioris A.N., Vardas P.E. (2002). Plasma Levels of Nitrites/Nitrates in Patients with Chronic Atrial Fibrillation are Increased after Electrical Restoration of Sinus Rhythm. J. Interv. Card. Electrophysiol. Int. J. Arrhythm. Pacing.

[77] Goette A., Hammwohner M., Bukowska A., Scalera F., Martens-Lobenhoffer J., Dobrev D., Ravens U., Weinert S., Medunjanin S., Lendeckel U. (2012). The impact of rapid atrial pacing on ADMA and endothelial NOS. Int. J. Cardiol..

[78] Liu H., Qu X., Liang Z., Chen W., Xia W., Song Y. (2008). Variance of DDAH/PRMT/ADMA pathway in atrial fibrillation dogs. Biochem. Biophys. Res. Commun..

[79] Feng Q., Lu X., Fortin A.J., Pettersson A., Hedner T., Kline R.L., Arnold J.M. (1998). Elevation of an endogenous inhibitor of nitric oxide synthesis in experimental congestive heart failure. Cardiovasc. Res..

[80] Saitoh M., Osanai T., Kamada T., Matsunaga T., Ishizaka H., Hanada H., Okumura K. (2003). High plasma level of asymmetric dimethylarginine in patients with acutely exacerbated congestive heart failure: Role in reduction of plasma nitric oxide level. Heart Vessel..

[81] Yamashita T., Sekiguchi A., Kato T., Tsuneda T., Iwasaki Y.K., Sagara K., Iinuma H., Sawada H., Aizawa T. (2007). Angiotensin type 1 receptor blockade prevents endocardial dysfunction of rapidly paced atria in rats. J. Renin-Angiotensin-Aldosterone Syst. JRAAS.

[82] Kim H.S., No C.W., Goo S.H., Cha T.J. (2013). An Angiotensin receptor blocker prevents arrhythmogenic left atrial remodeling in a rat post myocardial infarction induced heart failure model. J. Korean Med. Sci..

[83] Bukowska A., Rocken C., Erxleben M., Rohl F.W., Hammwohner M., Huth C., Ebert M.P., Lendeckel U., Goette A. (2010). Atrial expression of endothelial nitric oxide synthase in patients with and without atrial fibrillation. Cardiovasc. Pathol. Off. J. Soc. Cardiovasc. Pathol..

[84] Kaludercic N., Carpi A., Nagayama T., Sivakumaran V., Zhu G., Lai E.W., Bedja D., De Mario A., Chen K., Gabrielson K.L. (2014). Monoamine oxidase B prompts mitochondrial and cardiac dysfunction in pressure overloaded hearts. Antioxid. Redox Signal..

[85] Kaludercic N., Takimoto E., Nagayama T., Feng N., Lai E.W., Bedja D., Chen K., Gabrielson K.L., Blakely R.D., Shih J.C. (2010). Monoamine oxidase A-mediated enhanced catabolism of norepinephrine contributes to adverse remodeling and pump failure in hearts with pressure overload. Circ. Res..

[86] Madamanchi N.R., Runge M.S. (2013). Redox signaling in cardiovascular health and disease. Free. Radic. Biol. Med..

[87] Purohit A., Rokita A.G., Guan X., Chen B., Koval O.M., Voigt N., Neef S., Sowa T., Gao Z., Luczak E.D. (2013). Oxidized Ca^2+^/calmodulin-dependent protein kinase II triggers atrial fibrillation. Circulation.

[88] Luczak E.D., Anderson M.E. (2014). CaMKII oxidative activation and the pathogenesis of cardiac disease. J. Mol. Cell. Cardiol..

[89] Neef S., Dybkova N., Sossalla S., Ort K.R., Fluschnik N., Neumann K., Seipelt R., Schondube F.A., Hasenfuss G., Maier L.S. (2010). CaMKII-dependent diastolic SR Ca^2+^ leak and elevated diastolic Ca^2+^ levels in right atrial myocardium of patients with atrial fibrillation. Circ. Res..

[90] Gao G., Dudley S.C. (2009). Redox regulation, NF-kappaB, and atrial fibrillation. Antioxid. Redox Signal..

[91] Shang L.L., Dudley S.C. (2005). Tandem promoters and developmentally regulated 5′- and 3′-mRNA untranslated regions of the mouse Scn5a cardiac sodium channel. J. Biol. Chem..

[92] Shang L.L., Sanyal S., Pfahnl A.E., Jiao Z., Allen J., Liu H., Dudley S.C. (2008). NF-kappaB-dependent transcriptional regulation of the cardiac scn5a sodium channel by angiotensin II. American journal of physiology. Cell Physiol..

[93] Bowie A., O’Neill L.A. (2000). Oxidative stress and nuclear factor-kappaB activation: A reassessment of the evidence in the light of recent discoveries. Biochem. Pharmacol..

[94] Dolmetsch R.E., Xu K., Lewis R.S. (1998). Calcium oscillations increase the efficiency and specificity of gene expression. Nature.

[95] Kunsch C., Medford R.M. (1999). Oxidative stress as a regulator of gene expression in the vasculature. Circ. Res..

[96] Burstein B., Libby E., Calderone A., Nattel S. (2008). Differential behaviors of atrial versus ventricular fibroblasts: A potential role for platelet-derived growth factor in atrial-ventricular remodeling differences. Circulation.

[97] Adam O., Lavall D., Theobald K., Hohl M., Grube M., Ameling S., Sussman M.A., Rosenkranz S., Kroemer H.K., Schafers H.J. (2010). Rac1-induced connective tissue growth factor regulates connexin 43 and N-cadherin expression in atrial fibrillation. J. Am. Coll. Cardiol..

[98] Sulciner D.J., Irani K., Yu Z.X., Ferrans V.J., Goldschmidt-Clermont P., Finkel T. (1996). rac1 regulates a cytokine-stimulated, redox-dependent pathway necessary for NF-kappaB activation. Mol. Cell. Biol..

[99] Yang D., Yuan J., Liu G., Ling Z., Zeng H., Chen Y., Zhang Y., She Q., Zhou X. (2013). Angiotensin receptor blockers and statins could alleviate atrial fibrosis via regulating platelet-derived growth factor/Rac1/nuclear factor-kappa B Axis. Int. J. Med. Sci..

[100] Lin C.C., Chiang L.L., Lin C.H., Shih C.H., Liao Y.T., Hsu M.J., Chen B.C. (2007). Transforming growth factor-beta1 stimulates heme oxygenase-1 expression via the PI3K/Akt and NF-kappaB pathways in human lung epithelial cells. Eur. J. Pharmacol..

[101] Tyrrell R. (1999). Redox regulation and oxidant activation of heme oxygenase-1. Free. Radic. Res..

[102] Ishii T., Itoh K., Takahashi S., Sato H., Yanagawa T., Katoh Y., Bannai S., Yamamoto M. (2000). Transcription factor Nrf2 coordinately regulates a group of oxidative stress-inducible genes in macrophages. J. Biol. Chem..

[103] Kiriakidis S., Andreakos E., Monaco C., Foxwell B., Feldmann M., Paleolog E. (2003). VEGF expression in human macrophages is NF-kappaB-dependent: Studies using adenoviruses expressing the endogenous NF-kappaB inhibitor IkappaBalpha and a kinase-defective form of the IkappaB kinase 2. J. Cell Sci..

[104] Martin D., Galisteo R., Gutkind J.S. (2009). CXCL8/IL8 stimulates vascular endothelial growth factor (VEGF) expression and the autocrine activation of VEGFR2 in endothelial cells by activating NFkappaB through the CBM (Carma3/Bcl10/Malt1) complex. J. Biol. Chem..

[105] Lawrence D.M., Seth P., Durham L., Diaz F., Boursiquot R., Ransohoff R.M., Major E.O. (2006). Astrocyte differentiation selectively upregulates CCL2/monocyte chemoattractant protein-1 in cultured human brain-derived progenitor cells. Glia.

[106] Bonello S., Zahringer C., BelAiba R.S., Djordjevic T., Hess J., Michiels C., Kietzmann T., Gorlach A. (2007). Reactive oxygen species activate the HIF-1alpha promoter via a functional NFkappaB site. Arterioscler. Thromb. Vasc. Biol..

[107] Qiu X.B., Shao Y.M., Miao S., Wang L. (2006). The diversity of the DnaJ/Hsp40 family, the crucial partners for Hsp70 chaperones. Cell. Mol. Life Sci. CMLS.

[108] Bloom D.A., Jaiswal A.K. (2003). Phosphorylation of Nrf2 at Ser40 by protein kinase C in response to antioxidants leads to the release of Nrf2 from INrf2, but is not required for Nrf2 stabilization/accumulation in the nucleus and transcriptional activation of antioxidant response element-mediated NAD(P)H:quinone oxidoreductase-1 gene expression. J. Biol. Chem..

[109] Wu X., Huang L., Liu J. (2021). Relationship between oxidative stress and nuclear factor-erythroid-2-related factor 2 signaling in diabetic cardiomyopathy (Review). Exp. Ther. Med..

[110] Cheng X., Ku C.H., Siow R.C. (2013). Regulation of the Nrf2 antioxidant pathway by microRNAs: New players in micromanaging redox homeostasis. Free. Radic. Biol. Med..

[111] Mori M.A., Raghavan P., Thomou T., Boucher J., Robida-Stubbs S., Macotela Y., Russell S.J., Kirkland J.L., Blackwell T.K., Kahn C.R. (2012). Role of microRNA processing in adipose tissue in stress defense and longevity. Cell Metab..

[112] Ungvari Z., Tucsek Z., Sosnowska D., Toth P., Gautam T., Podlutsky A., Csiszar A., Losonczy G., Valcarcel-Ares M.N., Sonntag W.E. (2013). Aging-induced dysregulation of dicer1-dependent microRNA expression impairs angiogenic capacity of rat cerebromicrovascular endothelial cells. J. Gerontol. Ser. A Biol. Sci. Med Sci..

[113] Wiesen J.L., Tomasi T.B. (2009). Dicer is regulated by cellular stresses and interferons. Mol. Immunol..

[114] Santulli G., Iaccarino G., De Luca N., Trimarco B., Condorelli G. (2014). Atrial fibrillation and microRNAs. Front. Physiol..

[115] Liang Y., Ridzon D., Wong L., Chen C. (2007). Characterization of microRNA expression profiles in normal human tissues. BMC Genom..

[116] Lu Y., Zhang Y., Wang N., Pan Z., Gao X., Zhang F., Zhang Y., Shan H., Luo X., Bai Y. (2010). MicroRNA-328 contributes to adverse electrical remodeling in atrial fibrillation. Circulation.

[117] Wang Z., Lu Y., Yang B. (2011). MicroRNAs and atrial fibrillation: New fundamentals. Cardiovasc. Res..

[118] Liu H., Chen G.X., Liang M.Y., Qin H., Rong J., Yao J.P., Wu Z.K. (2014). Atrial fibrillation alters the microRNA expression profiles of the left atria of patients with mitral stenosis. BMC Cardiovasc. Disord..

[119] Liu H., Qin H., Chen G.X., Liang M.Y., Rong J., Yao J.P., Wu Z.K. (2014). Comparative expression profiles of microRNA in left and right atrial appendages from patients with rheumatic mitral valve disease exhibiting sinus rhythm or atrial fibrillation. J. Transl. Med..

[120] Roldan V., Arroyo A.B., Salloum-Asfar S., Manzano-Fernandez S., Garcia-Barbera N., Marin F., Vicente V., Gonzalez-Conejero R., Martinez C. (2014). Prognostic role of MIR146A polymorphisms for cardiovascular events in atrial fibrillation. Thromb. Haemost..

[121] Kimura W., Sadek H.A. (2012). The cardiac hypoxic niche: Emerging role of hypoxic microenvironment in cardiac progenitors. Cardiovasc. Diagn. Ther..

[122] Arany Z., Foo S.Y., Ma Y., Ruas J.L., Bommi-Reddy A., Girnun G., Cooper M., Laznik D., Chinsomboon J., Rangwala S.M. (2008). HIF-independent regulation of VEGF and angiogenesis by the transcriptional coactivator PGC-1alpha. Nature.

[123] Ferrara N., Frantz G., LeCouter J., Dillard-Telm L., Pham T., Draksharapu A., Giordano T., Peale F. (2003). Differential expression of the angiogenic factor genes vascular endothelial growth factor (VEGF) and endocrine gland-derived VEGF in normal and polycystic human ovaries. Am. J. Pathol..

[124] Adam O., Theobald K., Lavall D., Grube M., Kroemer H.K., Ameling S., Schafers H.J., Bohm M., Laufs U. (2011). Increased lysyl oxidase expression and collagen cross-linking during atrial fibrillation. J. Mol. Cell. Cardiol..

[125] Hornstra I.K., Birge S., Starcher B., Bailey A.J., Mecham R.P., Shapiro S.D. (2003). Lysyl oxidase is required for vascular and diaphragmatic development in mice. J. Biol. Chem..

[126] Maki J.M., Rasanen J., Tikkanen H., Sormunen R., Makikallio K., Kivirikko K.I., Soininen R. (2002). Inactivation of the lysyl oxidase gene Lox leads to aortic aneurysms, cardiovascular dysfunction, and perinatal death in mice. Circulation.

[127] Rodriguez C., Alcudia J.F., Martinez-Gonzalez J., Raposo B., Navarro M.A., Badimon L. (2008). Lysyl oxidase (LOX) down-regulation by TNFalpha: A new mechanism underlying TNFalpha-induced endothelial dysfunction. Atherosclerosis.

[128] Smith-Mungo L.I., Kagan H.M. (1998). Lysyl oxidase: Properties, regulation and multiple functions in biology. Matrix Biol. J. Int. Soc. Matrix Biol..

[129] Li W., Nellaiappan K., Strassmaier T., Graham L., Thomas K.M., Kagan H.M. (1997). Localization and activity of lysyl oxidase within nuclei of fibrogenic cells. Proc. Natl. Acad. Sci. USA.

[130] Rodriguez C., Martinez-Gonzalez J., Raposo B., Alcudia J.F., Guadall A., Badimon L. (2008). Regulation of lysyl oxidase in vascular cells: Lysyl oxidase as a new player in cardiovascular diseases. Cardiovasc. Res..

[131] Lazarus H.M., Cruikshank W.W., Narasimhan N., Kagan H.M., Center D.M. (1995). Induction of human monocyte motility by lysyl oxidase. Matrix Biol. J. Int. Soc. Matrix Biol..

[132] Li W., Liu G., Chou I.N., Kagan H.M. (2000). Hydrogen peroxide-mediated, lysyl oxidase-dependent chemotaxis of vascular smooth muscle cells. J. Cell. Biochem..

[133] Goette A. (2021). Is left atrial strain the pathophysiological link between transplanted stem cells and atrial fibrillation?. Int. J. Cardiol..

[134] Goette A., Jentsch-Ullrich K., Lendeckel U., Rocken C., Agbaria M., Auricchio A., Mohren M., Franke A., Klein H.U. (2003). Effect of atrial fibrillation on hematopoietic progenitor cells: A novel pathophysiological role of the atrial natriuretic peptide?. Circulation.

[135] Galea R., Cardillo M.T., Caroli A., Marini M.G., Sonnino C., Narducci M.L., Biasucci L.M. (2014). Inflammation and C-reactive protein in atrial fibrillation: Cause or effect?. Tex. Heart Inst. J..

[136] Suthahar N., Meijers W.C., Sillje H.H.W., de Boer R.A. (2017). From Inflammation to Fibrosis-Molecular and Cellular Mechanisms of Myocardial Tissue Remodeling and Perspectives on Differential Treatment Opportunities. Curr. Heart Fail. Rep..

[137] Vonderlin N., Siebermair J., Kaya E., Kohler M., Rassaf T., Wakili R. (2019). Critical inflammatory mechanisms underlying arrhythmias. Herz.

[138] Frustaci A., Chimenti C., Bellocci F., Morgante E., Russo M.A., Maseri A. (1997). Histological substrate of atrial biopsies in patients with lone atrial fibrillation. Circulation.

[139] Lee S.H., Chen Y.C., Chen Y.J., Chang S.L., Tai C.T., Wongcharoen W., Yeh H.I., Lin C.I., Chen S.A. (2007). Tumor necrosis factor-alpha alters calcium handling and increases arrhythmogenesis of pulmonary vein cardiomyocytes. Life Sci..

[140] Wakili R., Voigt N., Kaab S., Dobrev D., Nattel S. (2011). Recent advances in the molecular pathophysiology of atrial fibrillation. J. Clin. Investig..

[141] Yao C., Veleva T., Scott L., Cao S., Li L., Chen G., Jeyabal P., Pan X., Alsina K.M., Abu-Taha I.D. (2018). Enhanced Cardiomyocyte NLRP3 Inflammasome Signaling Promotes Atrial Fibrillation. Circulation.

[142] Kim N., Jung Y., Nam M., Sun Kang M., Lee M.K., Cho Y., Choi E.K., Hwang G.S., Soo Kim H. (2017). Angiotensin II affects inflammation mechanisms via AMPK-related signalling pathways in HL-1 atrial myocytes. Sci. Rep..

[143] Goette A., Lendeckel U. (2008). Electrophysiological effects of angiotensin II. Part I: Signal transduction and basic electrophysiological mechanisms. EP Eur..

[144] Psychari S.N., Apostolou T.S., Sinos L., Hamodraka E., Liakos G., Kremastinos D.T. (2005). Relation of elevated C-reactive protein and interleukin-6 levels to left atrial size and duration of episodes in patients with atrial fibrillation. Am. J. Cardiol..

[145] Aulin J., Siegbahn A., Hijazi Z., Ezekowitz M.D., Andersson U., Connolly S.J., Huber K., Reilly P.A., Wallentin L., Oldgren J. (2015). Interleukin-6 and C-reactive protein and risk for death and cardiovascular events in patients with atrial fibrillation. Am. Heart J..

[146] Wazni O., Martin D.O., Marrouche N.F., Shaaraoui M., Chung M.K., Almahameed S., Schweikert R.A., Saliba W.I., Natale A. (2005). C reactive protein concentration and recurrence of atrial fibrillation after electrical cardioversion. Heart.

[147] Jiang Z., Dai L., Song Z., Li H., Shu M. (2013). Association between C-reactive protein and atrial fibrillation recurrence after catheter ablation: A meta-analysis. Clin. Cardiol..

[148] Yo C.H., Lee S.H., Chang S.S., Lee M.C., Lee C.C. (2014). Value of high-sensitivity C-reactive protein assays in predicting atrial fibrillation recurrence: A systematic review and meta-analysis. BMJ Open.

[149] Schnabel R.B., Larson M.G., Yamamoto J.F., Sullivan L.M., Pencina M.J., Meigs J.B., Tofler G.H., Selhub J., Jacques P.F., Wolf P.A. (2010). Relations of biomarkers of distinct pathophysiological pathways and atrial fibrillation incidence in the community. Circulation.

[150] Tsang T.S., Barnes M.E., Miyasaka Y., Cha S.S., Bailey K.R., Verzosa G.C., Seward J.B., Gersh B.J. (2008). Obesity as a risk factor for the progression of paroxysmal to permanent atrial fibrillation: A longitudinal cohort study of 21 years. Eur. Heart J..

[151] Wanahita N., Messerli F.H., Bangalore S., Gami A.S., Somers V.K., Steinberg J.S. (2008). Atrial fibrillation and obesity—Results of a meta-analysis. Am. Heart J..

[152] Wang T.J., Parise H., Levy D., D’Agostino R.B., Wolf P.A., Vasan R.S., Benjamin E.J. (2004). Obesity and the risk of new-onset atrial fibrillation. JAMA.

[153] Watanabe H., Tanabe N., Watanabe T., Darbar D., Roden D.M., Sasaki S., Aizawa Y. (2008). Metabolic syndrome and risk of development of atrial fibrillation: The Niigata preventive medicine study. Circulation.

[154] Zacharias A., Schwann T.A., Riordan C.J., Durham S.J., Shah A.S., Habib R.H. (2005). Obesity and risk of new-onset atrial fibrillation after cardiac surgery. Circulation.

[155] Mahabadi A.A., Massaro J.M., Rosito G.A., Levy D., Murabito J.M., Wolf P.A., O’Donnell C.J., Fox C.S., Hoffmann U. (2009). Association of pericardial fat, intrathoracic fat, and visceral abdominal fat with cardiovascular disease burden: The Framingham Heart Study. Eur. Heart J..

[156] Rosito G.A., Massaro J.M., Hoffmann U., Ruberg F.L., Mahabadi A.A., Vasan R.S., O’Donnell C.J., Fox C.S. (2008). Pericardial fat, visceral abdominal fat, cardiovascular disease risk factors, and vascular calcification in a community-based sample: The Framingham Heart Study. Circulation.

[157] Al Chekakie M.O., Welles C.C., Metoyer R., Ibrahim A., Shapira A.R., Cytron J., Santucci P., Wilber D.J., Akar J.G. (2010). Pericardial fat is independently associated with human atrial fibrillation. J. Am. Coll. Cardiol..

[158] Tsao H.M., Hu W.C., Wu M.H., Tai C.T., Lin Y.J., Chang S.L., Lo L.W., Hu Y.F., Tuan T.C., Wu T.J. (2011). Quantitative analysis of quantity and distribution of epicardial adipose tissue surrounding the left atrium in patients with atrial fibrillation and effect of recurrence after ablation. Am. J. Cardiol..

[159] Schotten U., Verheule S., Kirchhof P., Goette A. (2011). Pathophysiological mechanisms of atrial fibrillation: A translational appraisal. Physiol. Rev..

[160] Goette A., Lendeckel U., Kuchenbecker A., Bukowska A., Peters B., Klein H.U., Huth C., Rocken C. (2007). Cigarette smoking induces atrial fibrosis in humans via nicotine. Heart.

[161] Venteclef N., Guglielmi V., Balse E., Gaborit B., Cotillard A., Atassi F., Amour J., Leprince P., Dutour A., Clement K. (2015). Human epicardial adipose tissue induces fibrosis of the atrial myocardium through the secretion of adipo-fibrokines. Eur. Heart J..

[162] Haemers P., Hamdi H., Guedj K., Suffee N., Farahmand P., Popovic N., Claus P., LePrince P., Nicoletti A., Jalife J. (2017). Atrial fibrillation is associated with the fibrotic remodeling of adipose tissue in the subepicardium of human and sheep atria. Eur. Heart J..

[163] Suffee N., Moore-Morris T., Jagla B., Mougenot N., Dilanian G., Berthet M., Proukhnitzky J., Le Prince P., Tregouet D.A., Puceat M. (2020). Reactivation of the Epicardium at the Origin of Myocardial Fibro-Fatty Infiltration During the Atrial Cardiomyopathy. Circ. Res..

[164] Goette A., Patscheke M., Henschke F., Hammwohner M. (2020). COVID-19-Induced Cytokine Release Syndrome Associated with Pulmonary Vein Thromboses, Atrial Cardiomyopathy, and Arterial Intima Inflammation. TH Open.

[165] Nattel S., Heijman J., Zhou L., Dobrev D. (2020). Molecular Basis of Atrial Fibrillation Pathophysiology and Therapy: A Translational Perspective. Circ. Res..

[166] Batal O., Schoenhagen P., Shao M., Ayyad A.E., Van Wagoner D.R., Halliburton S.S., Tchou P.J., Chung M.K. (2010). Left atrial epicardial adiposity and atrial fibrillation. Circ. Arrhythmia Electrophysiol..

[167] Nagashima K., Okumura Y., Watanabe I., Nakai T., Ohkubo K., Kofune M., Mano H., Sonoda K., Hiro T., Nikaido M. (2012). Does location of epicardial adipose tissue correspond to endocardial high dominant frequency or complex fractionated atrial electrogram sites during atrial fibrillation?. Circ. Arrhythmia Electrophysiol..

[168] Shin S.Y., Yong H.S., Lim H.E., Na J.O., Choi C.U., Choi J.I., Kim S.H., Kim J.W., Kim E.J., Park S.W. (2011). Total and interatrial epicardial adipose tissues are independently associated with left atrial remodeling in patients with atrial fibrillation. J. Cardiovasc. Electrophysiol..

[169] Hammwohner M., Bukowska A., Mahardika W., Goette A. (2019). Clinical importance of atrial cardiomyopathy. Int. J. Cardiol..

[170] Nakatani Y., Kumagai K., Minami K., Nakano M., Inoue H., Oshima S. (2015). Location of epicardial adipose tissue affects the efficacy of a combined dominant frequency and complex fractionated atrial electrogram ablation of atrial fibrillation. Heart Rhythm..

[171] Aviles R.J., Martin D.O., Apperson-Hansen C., Houghtaling P.L., Rautaharju P., Kronmal R.A., Tracy R.P., Van Wagoner D.R., Psaty B.M., Lauer M.S. (2003). Inflammation as a risk factor for atrial fibrillation. Circulation.

[172] Bruins P., te Velthuis H., Yazdanbakhsh A.P., Jansen P.G., van Hardevelt F.W., de Beaumont E.M., Wildevuur C.R., Eijsman L., Trouwborst A., Hack C.E. (1997). Activation of the complement system during and after cardiopulmonary bypass surgery: Postsurgery activation involves C-reactive protein and is associated with postoperative arrhythmia. Circulation.

[173] Mazurek T., Zhang L., Zalewski A., Mannion J.D., Diehl J.T., Arafat H., Sarov-Blat L., O’Brien S., Keiper E.A., Johnson A.G. (2003). Human epicardial adipose tissue is a source of inflammatory mediators. Circulation.

[174] Watanabe T., Takeishi Y., Hirono O., Itoh M., Matsui M., Nakamura K., Tamada Y., Kubota I. (2005). C-reactive protein elevation predicts the occurrence of atrial structural remodeling in patients with paroxysmal atrial fibrillation. Heart Vessel..

[175] Ozcan K.S., Gungor B., Altay S., Osmonov D., Ekmekci A., Ozpamuk F., Kemaloglu T., Yildirim A., Tayyareci G., Erdinler I. (2014). Increased level of resistin predicts development of atrial fibrillation. J. Cardiol..

[176] Gungor H., Ayik M.F., Kirilmaz B., Ertugay S., Gul I., Yildiz B.S., Nalbantgil S., Zoghi M. (2011). Serum resistin level: As a predictor of atrial fibrillation after coronary artery bypass graft surgery. Coron. Artery. Dis..

[177] Barakat B., Almeida M.E.F. (2021). Biochemical and immunological changes in obesity. Arch. Biochem. Biophys..

[178] Lin Y.K., Chen Y.C., Chen J.H., Chen S.A., Chen Y.J. (2012). Adipocytes modulate the electrophysiology of atrial myocytes: Implications in obesity-induced atrial fibrillation. Basic Res. Cardiol..

[179] Pantanowitz L. (2001). Fat infiltration in the heart. Heart.

[180] Samanta R., Pouliopoulos J., Thiagalingam A., Kovoor P. (2016). Role of adipose tissue in the pathogenesis of cardiac arrhythmias. Heart Rhythm..

[181] Huxley R.R., Filion K.B., Konety S., Alonso A. (2011). Meta-analysis of cohort and case-control studies of type 2 diabetes mellitus and risk of atrial fibrillation. Am. J. Cardiol..

[182] da Silva R.M. (2017). Influence of Inflammation and Atherosclerosis in Atrial Fibrillation. Curr. Atheroscler. Rep..

[183] Gutierrez A., Van Wagoner D.R. (2015). Oxidant and Inflammatory Mechanisms and Targeted Therapy in Atrial Fibrillation: An Update. J. Cardiovasc. Pharmacol..

[184] Karam B.S., Chavez-Moreno A., Koh W., Akar J.G., Akar F.G. (2017). Oxidative stress and inflammation as central mediators of atrial fibrillation in obesity and diabetes. Cardiovasc. Diabetol..

[185] Van Wagoner D.R. (2008). Oxidative stress and inflammation in atrial fibrillation: Role in pathogenesis and potential as a therapeutic target. J. Cardiovasc. Pharmacol..

[186] Anderson E.J., Kypson A.P., Rodriguez E., Anderson C.A., Lehr E.J., Neufer P.D. (2009). Substrate-specific derangements in mitochondrial metabolism and redox balance in the atrium of the type 2 diabetic human heart. J. Am. Coll. Cardiol..

[187] Zhang X., Zhang Z., Zhao Y., Jiang N., Qiu J., Yang Y., Li J., Liang X., Wang X., Tse G. (2017). Alogliptin, a Dipeptidyl Peptidase-4 Inhibitor, Alleviates Atrial Remodeling and Improves Mitochondrial Function and Biogenesis in Diabetic Rabbits. J. Am. Heart Assoc..

[188] Shan J., Xie W., Betzenhauser M., Reiken S., Chen B.X., Wronska A., Marks A.R. (2012). Calcium leak through ryanodine receptors leads to atrial fibrillation in 3 mouse models of catecholaminergic polymorphic ventricular tachycardia. Circ. Res..

[189] Cavalera M., Wang J., Frangogiannis N.G. (2014). Obesity, metabolic dysfunction, and cardiac fibrosis: Pathophysiological pathways, molecular mechanisms, and therapeutic opportunities. Transl. Res..

[190] Zhao L.M., Zhang W., Wang L.P., Li G.R., Deng X.L. (2012). Advanced glycation end products promote proliferation of cardiac fibroblasts by upregulation of KCa3.1 channels. Pflügers Arch. Eur. J. Physiol..

[191] Oldfield M.D., Bach L.A., Forbes J.M., Nikolic-Paterson D., McRobert A., Thallas V., Atkins R.C., Osicka T., Jerums G., Cooper M.E. (2001). Advanced glycation end products cause epithelial-myofibroblast transdifferentiation via the receptor for advanced glycation end products (RAGE). J. Clin. Investig..

[192] Goette A., Arndt M., Rocken C., Spiess A., Staack T., Geller J.C., Huth C., Ansorge S., Klein H.U., Lendeckel U. (2000). Regulation of angiotensin II receptor subtypes during atrial fibrillation in humans. Circulation.

[193] Goette A., Bukowska A., Lendeckel U., Erxleben M., Hammwohner M., Strugala D., Pfeiffenberger J., Rohl F.W., Huth C., Ebert M.P. (2008). Angiotensin II receptor blockade reduces tachycardia-induced atrial adhesion molecule expression. Circulation.

[194] Goette A., Lendeckel U., Klein H.U. (2002). Signal transduction systems and atrial fibrillation. Cardiovasc. Res..

[195] Goette A., Staack T., Rocken C., Arndt M., Geller J.C., Huth C., Ansorge S., Klein H.U., Lendeckel U. (2000). Increased expression of extracellular signal-regulated kinase and angiotensin-converting enzyme in human atria during atrial fibrillation. J. Am. Coll. Cardiol..

[196] Barton M., Carmona R., Ortmann J., Krieger J.E., Traupe T. (2003). Obesity-associated activation of angiotensin and endothelin in the cardiovascular system. Int. J. Biochem. Cell Biol..

[197] Frigolet M.E., Torres N., Tovar A.R. (2013). The renin-angiotensin system in adipose tissue and its metabolic consequences during obesity. J. Nutr. Biochem..

[198] Umemura S., Nyui N., Tamura K., Hibi K., Yamaguchi S., Nakamaru M., Ishigami T., Yabana M., Kihara M., Inoue S. (1997). Plasma angiotensinogen concentrations in obese patients. Am. J. Hypertens..

[199] Ailhaud G. (2006). Adipose tissue as a secretory organ: From adipogenesis to the metabolic syndrome. Comptes Rendus Biol..

[200] Fuentes P., Acuna M.J., Cifuentes M., Rojas C.V. (2010). The anti-adipogenic effect of angiotensin II on human preadipose cells involves ERK1,2 activation and PPARG phosphorylation. J. Endocrinol..

[201] Janke J., Schupp M., Engeli S., Gorzelniak K., Boschmann M., Sauma L., Nystrom F.H., Jordan J., Luft F.C., Sharma A.M. (2006). Angiotensin type 1 receptor antagonists induce human in-vitro adipogenesis through peroxisome proliferator-activated receptor-gamma activation. J. Hypertens..

[202] Jing F., Mogi M., Horiuchi M. (2013). Role of renin-angiotensin-aldosterone system in adipose tissue dysfunction. Mol. Cell Endocrinol..

[203] Furuhashi M., Ura N., Higashiura K., Murakami H., Tanaka M., Moniwa N., Yoshida D., Shimamoto K. (2003). Blockade of the renin-angiotensin system increases adiponectin concentrations in patients with essential hypertension. Hypertension.

[204] Mathai M.L., Naik S., Sinclair A.J., Weisinger H.S., Weisinger R.S. (2008). Selective reduction in body fat mass and plasma leptin induced by angiotensin-converting enzyme inhibition in rats. Int. J. Obes..

[205] Santos E.L., de Picoli Souza K., da Silva E.D., Batista E.C., Martins P.J., D’Almeida V., Pesquero J.B. (2009). Long term treatment with ACE inhibitor enalapril decreases body weight gain and increases life span in rats. Biochem. Pharmacol..

[206] Tian F., Luo R., Zhao Z., Wu Y., Ban D. (2010). Blockade of the RAS increases plasma adiponectin in subjects with metabolic syndrome and enhances differentiation and adiponectin expression of human preadipocytes. Exp. Clin. Endocrinol. Diabetes.

[207] Weisinger R.S., Stanley T.K., Begg D.P., Weisinger H.S., Spark K.J., Jois M. (2009). Angiotensin converting enzyme inhibition lowers body weight and improves glucose tolerance in C57BL/6J mice maintained on a high fat diet. Physiol. Behav..

[208] Furuhashi M., Ura N., Takizawa H., Yoshida D., Moniwa N., Murakami H., Higashiura K., Shimamoto K. (2004). Blockade of the renin-angiotensin system decreases adipocyte size with improvement in insulin sensitivity. J. Hypertens..

[209] Bukowska A., Schild L., Bornfleth P., Peter D., Wiese-Rischke C., Gardemann A., Isermann B., Walles T., Goette A. (2020). Activated clotting factor X mediates mitochondrial alterations and inflammatory responses via protease-activated receptor signaling in alveolar epithelial cells. Eur. J. Pharmacol..

[210] Bukowska A., Hammwohner M., Corradi D., Mahardhika W., Goette A. (2018). Atrial thrombogenesis in atrial fibrillation: Results from atrial fibrillation models and AF-patients. Herzschrittmacherther. Elektrophysiol..

[211] Fabritz L., Crijns H., Guasch E., Goette A., Hausler K.G., Kotecha D., Lewalter T., Meyer C., Potpara T.S., Rienstra M. (2021). Dynamic risk assessment to improve quality of care in patients with atrial fibrillation: The 7th AFNET/EHRA Consensus Conference. EP Eur..

[212] Schnabel R.B., Camen S., Knebel F., Hagendorff A., Bavendiek U., Bohm M., Doehner W., Endres M., Groschel K., Goette A. (2021). Expert opinion paper on cardiac imaging after ischemic stroke. Clin. Res. Cardiol..

[213] Goette A., Eckardt L., Valgimigli M., Lewalter T., Laeis P., Reimitz P.E., Smolnik R., Zierhut W., Tijssen J.G., Vranckx P. (2021). Clinical risk predictors in atrial fibrillation patients following successful coronary stenting: ENTRUST-AF PCI sub-analysis. Clin. Res. Cardiol..

[214] Goette A., Lip G.Y.H., Jin J., Heidbuchel H., Cohen A.A., Ezekowitz M., Merino J.L. (2020). Differences in Thromboembolic Complications Between Paroxysmal and Persistent Atrial Fibrillation Patients Following Electrical Cardioversion (From the ENSURE-AF Study). Am. J. Cardiol..

[215] Gorenek B., Pelliccia A., Benjamin E.J., Boriani G., Crijns H.J., Fogel R.I., Van Gelder I.C., Halle M., Kudaiberdieva G., Lane D.A. (2017). European Heart Rhythm Association (EHRA)/European Association of Cardiovascular Prevention and Rehabilitation (EACPR) position paper on how to prevent atrial fibrillation endorsed by the Heart Rhythm Society (HRS) and Asia Pacific Heart Rhythm Society (APHRS). EP Eur..

[216] Kotecha D., Breithardt G., Camm A.J., Lip G.Y.H., Schotten U., Ahlsson A., Arnar D., Atar D., Auricchio A., Bax J. (2018). Integrating new approaches to atrial fibrillation management: The 6th AFNET/EHRA Consensus Conference. EP Eur..

[217] Rocken C., Peters B., Juenemann G., Saeger W., Klein H.U., Huth C., Roessner A., Goette A. (2002). Atrial amyloidosis: An arrhythmogenic substrate for persistent atrial fibrillation. Circulation.

